# On the Orientation-Induced Crystallization of Polymers

**DOI:** 10.3390/polym8060229

**Published:** 2016-06-08

**Authors:** Koh-hei Nitta

**Affiliations:** Division of Natural System, Institute of Science and Engineering, Kanazawa University, Kakuma Campus, Kanazawa 920-1192, Japan; nitta@se.kanazawa-u.ac.jp; Tel.: +81-76-234-4184; Fax: +81-76-264-6220

**Keywords:** orientation-induced crystallization, crystalline polymers, hydrogen molecule ion

## Abstract

In order to understand orientation-induced crystallization of polymers, we introduced an intermolecular interaction between polymer chains based on quantum mechanics. We therefore considered a pair of perfectly extended chains where the intermolecular interaction is assumed to be based on the hydrogen interaction with a single chain. When two protons of each extended chain become closer together under tension, the attractive force between the extended chains is caused by the interaction between hydrogen atoms surrounding the main chains based on the hydrogen molecule ion H${}_{2}^{+}$. The energy is split into the ground and excited states, and the spontaneous process leading to the ground state is the origin for orientation-induced crystallization.

## 1. Introduction

**Figure 1:**
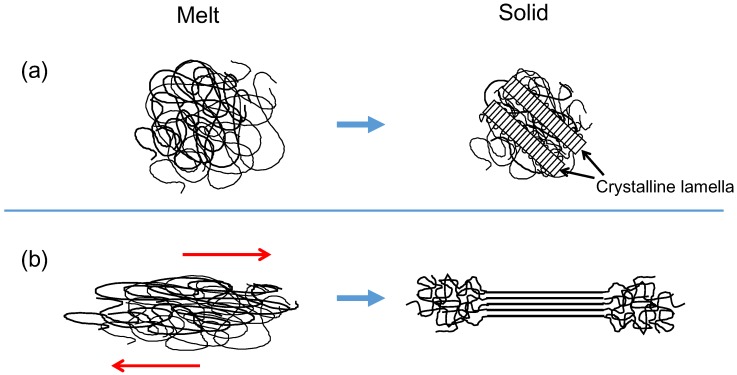
Schematic illustrations of crystallization from (**a**) an equilibrium melt and (**b**) a flowing melt.

Polymers are chain-like molecules made up of repeating units of a particular molecular group jointed together by covalent bonds [1]. One of the simplest polymers is polyethylene, which has a repeating unit of –CH${}_{2}$–CH${}_{2}$–. The basic unit of this sequence is called the “structural unit”, and the total number of the units in a molecule corresponds to the polymerization degree. When a polymer with configurational regularity is quenched to an ambient temperature from the melt, it undergoes a first-order phase transition from a disordered amorphous state to form an ordered crystalline structure [2,3], and they appear not to follow the Gibbs phase rule. Such a melt-crystallized polymer exhibits an alternating two-phase structure that consists of plate-like crystalline lamellae and the amorphous layers. The contour length of a polymer molecule is far greater than the typical lamellar thickness of the order of 10 nm, resulting in the formation of folded chain crystallites consisting of partially stretched conformations, and the chain axes within the lamellae are approximately normal to the face of the lamellae (see Figure 1a). Although the crystallizable arrangement of polymer chains has a lower conformational energy, concomitant reduction of the entropy required for the persistence of sequential crystalline conformation limits the crystalline lamellar thickness.

The molecular relaxation time of polymers is very long when compared with small molecules because of chain–chain entanglements in the melt, leading to high viscosity [4,5]. These slow relaxation dynamics impose kinetic activation barriers for the crystallization nucleation process under cooling. Accordingly, there exists a large amount of experimental kinetic data concerning nucleation and growth for polymer crystallization [6,7]. In addition, most theories of polymer isothermal crystallization from the melt state have been proposed based on the primary and secondary (or growth) nucleation kinetics [8,9,10,11,12,13].

A majority of commodity plastic products are manufactured by extrusion and injection molding from flowing melts followed by a crystallization process [14,15,16]. In the case of extrusion molding, the formation of oriented shish structures occurs in regions of high shear near the walls in an extrusion flow [17]. The layer nearest to the wall is the oriented skin, followed by a somewhat oriented fine-grained layer and finally isotropic structural morphology around the center of the die in the regions that experience no stress [17,18]. Regarding the manufacturing of polymer films and fibers, solid state drawing is usually carried out by a high speed drawing under flowing melt conditions. The structure and properties of these polymeric products depend on the manner in which polymer molecules crystallize in the drawing solid and/or flowing melt (see Figure 1b) [19].

When the draw rate is greater than the inverse of the chain retraction time, *i.e.*, the Weissenberg number is greater than unity, the orientation induced by stretching is not dissipated by viscous processes but is set as the polymer crystallizes after the draw process [20,21,22]. Draw-induced crystallization apparently proceeds without an activation barrier or a nucleus, and is limited only by local molecular relaxation [17,23]. This orientation-induced barrier-free transition to the crystalline phase is initiated by the spontaneous ordering of flexible conformations into rigid conformational sequences. The bundles of rigid sequence persist to temperatures higher than the nominal melting point. Aside from this, when natural rubbers are stretched beyond a critical value, chain molecules arrange themselves in an ordered structure, presumably composed of fibrillar chains aligned with the stretching direction, which is accompanied by the stress reduction [24,25,26]. This behavior is called the strain-induced crystallization, but the crystalline structure in stretched rubbers is found to start to melt as soon as retraction begins.

Consequently, a number of theoretical as well as computational investigations of orientation-induced crystallization have also been performed for material design in plastic products, films, fiber spinning, and rubbers so far [27,28,29,30]. It has been accepted that the orientation-induced crystallization mechanism from the sheared melt is quite different from that of the isothermal crystallization from the quiescent melt state. However, fundamental questions remain as to how chain-like molecules are crystallized under the drawing process. The aim of this study is to provide a physical insight into the origin of these orientation-induced crystallization processes. For that purpose, a pair of perfectly aligned extended chains are employed as the simplest model. A coupling interaction is investigated between hydrogen atoms surrounding the extended main chains from a quantum-mechanical point of view where van der Waals interections are not included semi-empirically.

## 2. Polymer Model

A polymer chain can take a huge number of different conformations in a melt state. This corresponds to micro-Brownian motion and the chains are rapidly converted into other conformations. Each conformation exists for only a very short time, so that the resulting conformations are temporal averages over all actual bonds. Time-averaged conformational states of the polymer can be calculated as ensemble averages over the successive steps of Brownian random walks. This results in polymer molecules being expressed by random flight chains composed of statistical bonds (effective bonds) joining beads of unit mass where an appropriate number of actual bonds are replaced by a single effective bond [31,32]. Real flexible polymers have been identified to be effectively treated as such a random flight chain, and the details of the chemical structure of real polymers can be smeared out [33,34]. This chain is a simple coarse-graining polymer chain [35].

According to the central limit theorem in statistical physics, the instantaneous shape of a linear chain, which is obtained by time-averaging over many conformations, can be described by a Gaussian distribution [36,37]. Here, let beads be labeled from numeral 1 though *N*. The distribution function $W(b_{i})$ of the effective bond vector $b_{i}=r_{i\;+\;1}-r_{i}$, where $r_{i}$ is the position vector of the *i*-th effective unit, can be defined either as the time-averaged incidence of $b_{i}$ within the specified range for a given molecule or as the average incidence for an ensemble of many identical units subject to identical conditions. Then, the distribution of the effective bond vector $b_{i}$ is given by a Gaussian distribution function [37]. Consequently, the probability distribution of the set of position vectors $\left\{r_{i}\right\}=\left\{r_{1},\ldots ,r_{N}\right\}$ is

(1) $$P\left(\left\{r_{i}\right\}\right)={\left(\frac{3}{2\pi \langle b^{2}\rangle }\right)}^{3/2}\exp\left[-\frac{3}{2\langle b^{2}\rangle }\sum_{i\;=\;1}^{N-1}{\left(r_{i\;+\;1}-r_{i}\right)}^{2}\right],$$

where $\langle b^{2}\rangle$ is the mean square of time-averaged bond length.

The equilibrium state of this chain is described by a distribution function proportional to exp(−V/kT), where *V* is the potential energy, *k* is the Boltzmann constant, and *T* is the absolute temperature. Therefore, if we choose

(2) $$V=\frac{3kT}{2\langle b^{2}\rangle }\sum_{i\;=\;1}^{N-1}{\left(r_{i\;+\;1}-r_{i}\right)}^{2},$$

then the chain’s equilibrium distribution function is reduced to Equation (1). This means that a polymer molecule can be modeled as a chain of beads connected by a Hookean spring with a spring constant $3kT/\langle b^{2}\rangle$ . Thus, the Hookean spring is associated with the dynamic potential based on the changes in conformational entropy [38].

The equilibrium distribution function of statistical bonds in random flight chains is consistent with the Gaussian distribution function if and only if the value of ${\langle b^{2}\rangle }^{1/2}$ is identical with the effective bond length *b*. The spring of the Gaussian chain is then called the “segment” [37], which is composed of several structural units. It follows that the probability density function for the end-to-end distance $R(=|r_{N}-r_{1}|)$ of a random flight chain with *N* beads can be expressed by

(3) $$P\left(R\right)=4\pi R^{2}{\left(\frac{3}{2\pi Nb^{2}}\right)}^{3/2}\exp\left(-\frac{3R^{2}}{2Nb^{2}}\right).$$

We can find that the root mean square of the end-to-end distance is $\sqrt{N}b$.

In this work, we accepted as an axiom that crystallizable polymers are flexible, and the flexible polymer chains before crystallization are expressed by a random flight chain composed of identical bonds (or segments) of length *b* joining *N* beads of unit mass (Axiom I). Because all conformations maintain the same internal energy during elongation under isothermal conditions, the free energy change ΔF for a single chain stretching process is dominated by the entropy change due to the extension of the chain through the conformational arrangement; *i.e.*, ΔF=−TΔs. The loss of conformational entropy is caused by changes in the number of bond arrangements.

**Figure 2:**
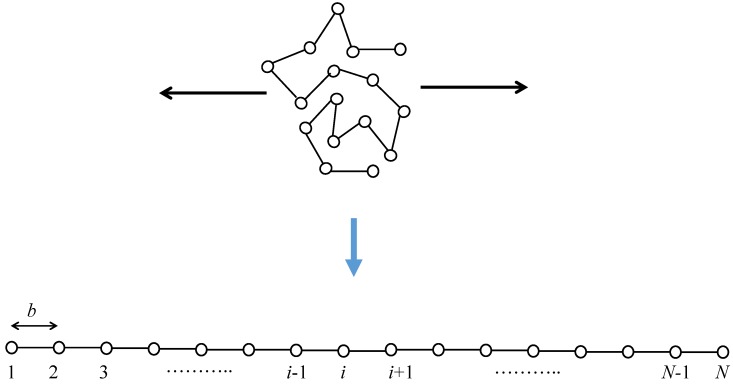
Schematic of stretching of a single random flight chain. Stretching is along the horizontal direction.

The set of conformation arrangements of the random flight chain can be considered to be the set of random walks of *N* steps with a step length of *b* in an appropriate coordinate system [39,40]. The total number of bond arrangements for the one-polymer system can be estimated to be $\Omega \left(N\right)=z^{N}$, where *z* is the number of possible microscopic state per each segment. The total number of random walk chains with *N* steps lying at an end-to-end distance being between *R* and R+dR is given by $z^{N}P\left(R\right)dR$. Consequently, the entropy change between the fully stretched state and the initial random coil state is estimated to be at most Δs=−Nklnz because the entropy of the fully stretched state is negligible. Figure 2 shows a schematic of the conformational change of a random flight chain from the initial random coil state to a completely oriented state. The mean end-to-end distance $\sqrt{N}b$ in the random coiling state increases up to Nb for the fully stretched state.

The chain conformational problem for random flight chains can be reduced in a highly oriented stage to that of a rotational isomeric state (RIS) model [34] by introducing a detailed mathematical description of the local chain structure in which we put z≅3 or more discrete values for conformations corresponding to the potential minima (e.g., tans and ±gauche), and we consider *N* to be the degree of polymerization. Notice that the mathematical description of RIS can be obtained on the basis of a familiar one-dimensional Ising problem [33].

## 3. Coupled Chains System

When crystallized polymers are uniaxially stretched under the appropriate tensile speed below their melting temperatures, a large scale of morphological transformation from isotropic to highly oriented or fibrillar structures takes place [41,42,43,44]. The ultimately extended-chain structure locally appears in the strain-hardening stage under uniaxial tension or in the cooling stage from sheared melt. The tensile load leads to the almost completely extended state from the isotropic random coil state in the initial stage, and the further loading to the system contracts the distance between the extended chains. This is called the “Poisson contraction” [45]. Consequently, the orientation-induced crystallization in chain molecules is likely to result from contributions of the intramolecular and intermolecular interactions [46].

**Figure 3:**
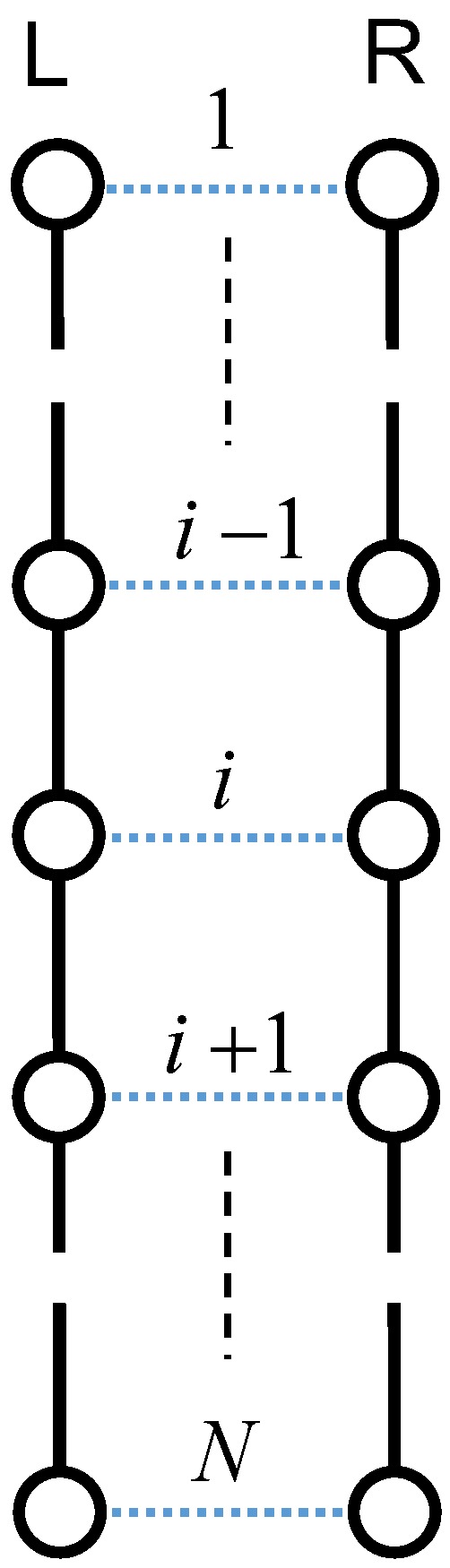
Schematic array of two extended chains with *N* units. Solid lines denote the C−C interaction, and the dotted lines denote the H⋯H interaction. Stretching is along the vertical direction.

To elucidate the mechanism of orientation-induced crystallization, we assumed the condition that the Weissenberg number is greater than unity at a fixed temperature below melting point. Here, we consider a pair of random flight chains consisting of *N* units of identical mass, jointed together by segments on the basis of Axiom I. The tensile loading to the system extends the random flight chains in the stretching direction, leading to a parallel alignment of two extended chains (see Figure 3) [47]. The left chain is denoted the L-chain and the right one is denoted the R-chain in the figure. Most polymer chains consist of a main carbon atom backbone saturated by covalently-bound hydrogen atoms [48,49]. Thus, the dominant intermolecular interactions are (H⋯H) interactions between the neighboring aligned chains, and the intramolecular interactions of the main chain are based on carbon–carbon covalent (C−C) interactions. In addition, we assume there are no interactions between adjacent protons of the same polymer chains.

In general, the ends of polymer chains are actually unlikely to be directly subjected to external forces because the span between two clamps of tensile machines is far greater than the end-to-end distance of single chains. The present coupled-chains model is assumed to represent a local part of specimen far enough from the location of load application or the clamps. Here, we employ the Saint–Venant’s principle [50] as the Axiom II and assert that the Poisson contraction is preserved throughout tension [45]. These mean that the external load is homogeneously dispersed over the L- and R-chains and that a pair of extended chains, after aligning to the stretching direction, are closer together perpendicular to the stretching direction according to the uniaxial elongation, being accompanied without bond stretching and angle bending. This is because the force constants of bond stretching and angle bending of main chains are considerably greater than that of interatomic interaction.

When two protons of the L- and R-chains are closer with a distance $r_{0}$, but not too large of a separation, the electron density around a proton bonded to a main chain carbon is spread out because of the uncertainty principle and interact with neighboring protons. For simplicity, we assume only interatomic interaction between the pair of protons of L- and R-chains, and assume that a half-electron brought by the L-proton is combined with another half-electron brought by the R-proton into a single electron, found half-way between each proton pair [47].

Consequently, *N* pairs of protons with a single electron are formed. It is well known that the imaginary hydrogen molecule ion H${}_{2}^{+}$ possesses a bound state at the minimum energy point; *i.e.*, a ground state whose energy is less than that of a hydrogen atom or a free proton combined. It should be noted here that the interaction system possesses the bonding orbital that has a possibility to cause association through interatomic bonding.

## 4. Orientation-Induced Crystallization Process

We take time zero, t=0, to be the time when two protons of extended chains are closer with a distance $r_{0}$, where their quantum atomic interaction is initiated. Since the Poisson contraction motion of protons is much slower than electron motions, electron and proton motions are decoupled and the interactions can be determined under the Born–Oppenheimer approximation [51]. The Hamiltonian of a proton pair with a single electron, *i.e.*, a hydrogen molecule ion H${}_{2}^{+}$ system, has the form

(4) $$H=-\frac{\hbar^{2}}{2m_{e}}\nabla^{2}-\frac{e^{2}}{r_{L}}-\frac{e^{2}}{r_{R}}+\frac{e^{2}}{r},$$

where ℏ(=h/2π) is the reduced Plank constant, *e* is the elementary charge in Gaussian unit, $m_{e}$ is the electron mass, *r* is the interproton distance, and $r_{R}$ and $r_{L}$ are the distainces of the electron from the L- and R-protons, respectively. Distances are given in atomic units (a.u.).

Here, we consider two symmetric states in which the single electron is trapped by either the L- or R-proton. We take these two different configurations as the base states, and we call them $|L\rangle$ and |R〉. Both can be considered to be one hydrogen atom in its ground state. Then, the two configurations are related by mirror reflection in the plane of a single proton pair and the expectation value of the energy α(r) is the same: $\alpha \left(r\right)=\langle L\;|\;H\;|\;L\rangle =\langle R\;|\;H\;|\;R\rangle$, which corresponds to be the ground-state energy of a hydrogen atom. Since two protons get close to one another as deformation proceeds, the electron jumps from one proton to the other. The exchange energy β(r) for the electron is given by $\beta \left(r\right)=\langle L\;|\;H\;|\;R\rangle =\langle R\;|\;H\;|\;L\rangle$.

The prerequisite [47] that a half-electron brought by the L-proton is combined with another half-electron brought by the R-proton into a single electron is here postulated. Then, letting the time-dependent of wave vector of the electron be |ψ(r)〉, we arrive at the following statement.

**Corollary 1.**  The probabilities of finding the electron around the L- and R-protons are the same at t=0: i.e., $|\langle L\;|\;\psi (0)\rangle {|}^{2}={\left|\langle R\;|\;\psi (0)\rangle \right|}^{2}=1/2$.

Any state $|\psi (r)\rangle$ at any t≥0 is represented by the linear combination of the two base vectors |L〉 and |R〉:

(5) $$|\psi (t)\rangle =|L\rangle \langle L\;|\;\psi (t)\rangle +|R\rangle \langle R\;|\;\psi (t)\rangle .$$

Under orthogonality conditions, $\langle L\;|\;L\rangle =\langle R\;|\;R\rangle =1$ and $\langle L\;|\;R\rangle =\langle R\;|\;L\rangle =0$. The amplitude vector satisfies the following time-dependent Schrödinger equation:

(6) $$i\hbar \frac{d}{dt}\left[\begin{array}{c} \langle L\;|\;\psi (t)\rangle \\ \langle R\;|\;\psi (t)\rangle \end{array}\right]=\left[\begin{array}{cc} \alpha (r) & -\beta (r)\\ -\beta (r) & \alpha (r) \end{array}\right]\cdot \left[\begin{array}{c} \langle L\;|\;\psi (t)\rangle \\ \langle R\;|\;\psi (t)\rangle \end{array}\right],$$

where $i=\sqrt{-1}$. We can determine the amplitudes $\langle L\;|\;\psi (t)\rangle$ and $\langle R\;|\;\psi (t)\rangle$ according to specific initial conditions. Their general solution is

(7) $$\left[\begin{array}{c} \langle L\;|\;\psi (t)\rangle \\ \langle R\;|\;\psi (t)\rangle \end{array}\right]=e^{-i\alpha (r)t/\hbar }\left[\begin{array}{cc} \cos\left(\beta (r)t/\hbar \right) & i\sin\left(\beta (r)t/\hbar \right)\\ i\sin\left(\beta (r)t/\hbar \right) & \cos\left(\beta (r)t/\hbar \right) \end{array}\right]\cdot \left[\begin{array}{c} \langle L\;|\;\psi (0)\rangle \\ \langle R\;|\;\psi (0)\rangle \end{array}\right].$$

Corollary 1 as the initial condition for the quantum interaction gives $\langle L\;|\;\psi (t)\rangle =\pm \langle R\;|\;\psi (t)\rangle$. Introducing this initial condition into Equation (7), the energy of the system is split into ground and excited states:

(8) $$\begin{array}{cccc} \varepsilon_{g} & =\alpha (r)+\beta (r), & \varepsilon_{u} & =\alpha (r)-\beta (r), \end{array}$$

where the α(r) and β(r) in Hartree units can be obtained as a function of a reduced distance *r* between protons using the Coulomb integral *J* and the resonance integral *K*:

(9) $$\alpha \left(r\right)=\varepsilon_{H}+\frac{1}{r}+J,$$

(10) β(r)=−K,

where $J=-1/r+\left(1+1/r\right)e^{-2r}$, $K=\left(1+r\right)e^{-r}$, and the $\varepsilon_{H}$ denotes the ground state energy of a hydrogen atom, which is −1/2 Hartree, and *r* is the interproton distance in atomic units. These equations are obtained under the orthogonally condition $\langle L\;|\;R\rangle =\langle R\;|\;L\rangle =0$. The eigenstates that have these definite energies have the form:

(11) $$\begin{array}{cccc} |E_{g}\rangle & =\frac{1}{\sqrt{2}}\left(|L\rangle +|R\rangle \right), & |E_{u}\rangle & =\frac{1}{\sqrt{2}}\left(|L\rangle -|R\rangle \right). \end{array}$$

**Figure 4:**
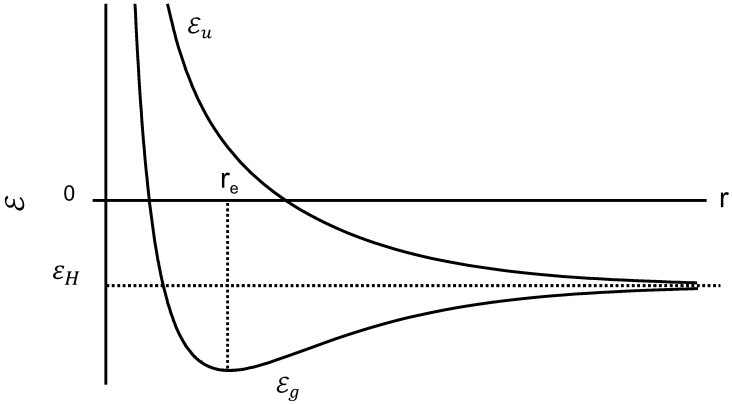
Bonding energy $\varepsilon_{g}$ and anti-bonding energy $\varepsilon_{u}$ plotted against the interproton distance.

The variation of the two energies $\varepsilon_{g}$ and $\varepsilon_{u}$ with the distance *r*$\left(<r_{0}\right)$ between two protons is shown in Figure 4.

**Proposition 2.**  The probability of finding the electron around the L-proton is the same with that of finding the electron around the R-proton being independent of time.

**Proof.**  When $|\psi (0)\rangle =|E_{g}\rangle$ at t=0, we have

(12) $$|\psi (t)\rangle =|E_{g}\rangle \exp\left(-i\frac{\varepsilon_{g}}{\hbar }t\right).$$

Likewise, when $|\psi (0)\rangle =|E_{u}\rangle$ at t=0, we have

(13) $$|\psi (t)\rangle =|E_{u}\rangle \exp\left(-i\frac{\varepsilon_{u}}{\hbar }t\right).$$

In both cases, we can confirm $|\langle L\;|\;\psi (t)\rangle {|}^{2}={\left|\langle R\;|\;\psi (t)\rangle \right|}^{2}=1/2$.  ☐

This proposition indicates that there exists only one electron between the L- and R-protons, and that electron has the same probability amplitude, $1/\sqrt{2}$, to be in either proton.

**Proposition 3.**  The probability for the system to take $\varepsilon_{g}$ or $\varepsilon_{u}$ is independent of time and is determined by the initial conditions.

**Proof.**  The two stationary states can be derived from Equation (12) or (13):

(14) $$\langle E_{g}\;|\;\psi (t)\rangle =\exp\left(-i\frac{\varepsilon_{g}}{\hbar }t\right)\langle E_{g}\;|\;\psi (0)\rangle ,$$

or

(15) $$\langle E_{u}\;|\;\psi (t)\rangle =\exp\left(-i\frac{\varepsilon_{u}}{\hbar }t\right)\langle E_{u}\;|\;\psi (0)\rangle .$$

Consequently, we have $|\langle E_{g}\;|\;\psi (t)\rangle {|}^{2}={\left|\langle E_{g}\;|\;\psi (0)\rangle \right|}^{2}$ or $|\langle E_{u}\;|\;\psi (t)\rangle {|}^{2}={\left|\langle E_{u}\;|\;\psi (0)\rangle \right|}^{2}$.  ☐

If the system is in the state $|E_{u}\rangle$, the energy increases as the distance between protons is decreased, and the quantum effects impart a repulsive force that tends to keep the protons apart. In contrast, the state $|E_{g}\rangle$ has a minimum energy point which is the equilibrium configuration and the lowest energy condition. The energy at this point is lower than the energy of a separated proton as shown in Figure 4. Both chains are spontaneously close at the minimum energy point $r=r_{e}$, coupled with the electron transition from the antibonding to the bonding orbital. Consequently, orientational crystallization may occur while the protons get close together, since the system becomes stable at $r=r_{e}$. The energy drop reflects the latent heat release, *i.e.*, Joule effect, being proportional to *N* where the value of *N* corresponds to the degree of crystallinity.

These propositions presented here are generalized in the quantum two-level system [52]. The above deductive inference leads us to the following theorem:

**Theorem 4.**  When a pair of two random flight chains representing typical flexible polymers surrounded by hydrogen atoms is homogeneously extended, a spontaneous alignment of the two perfectly extended chains is almost surely formed in the extending direction.

It should be noted here that the interproton interaction $r_{0}$ at t=0 cannot be determined with certainty, although the time of the onset of the quantum interproton interaction is set to be t=0. The uncertainty in the initial condition in addition to the onset of the electron transition may possibly lead to fluctuation of the onset time or position (strain) of crystallization.

## 5. Quantitative Analysis

To quantitatively estimate the energy profile between the L- and R-protons, we need to consider the non-orthogonality of the basis orbitals, where the overlap interaction $S=\langle R\;|\;L\rangle =\langle L\;|\;R\rangle \neq 0$ is positive [53,54]. The orbital function is assumed to be expressed as a linear combination of atomic orbitals, and their coefficients can be determined so as to minimize the total energy. The notable improvement in the energy profile when considering the non-orthogonality is an energy shift of the exchange integral, $\tilde{\beta }\left(r\right)=-K+\left(\varepsilon_{H}+1/r\right)S$, while the overall description of the energy profile under orthogonal conditions is maintained. Consequently, the two energy levels are adjusted by the overlap integral *S* as follows:

(16) $$\begin{array}{cccc} \varepsilon_{g} & =\frac{\alpha \left(r\right)+\tilde{\beta }\left(r\right)}{1+S}, & \varepsilon_{u} & =\frac{\alpha \left(r\right)-\tilde{\beta }\left(r\right)}{1-S}, \end{array}$$

where $S=\left(1+r+r^{2}/3\right)e^{-r}$.

Once *N* pairs of protons with a single electron are formed at t=0, the energy of each pair is then split into the ground and excited states, and the energy difference $2\delta \left(r\right)=\varepsilon_{u}-\varepsilon_{g}$ becomes larger as the chains are brought closer together. Let the electron transition to the ground state be allowed while both chains are aligned parallel to the stretching direction with a distance $r=r_{e}$. Then, the number of pairs in the ground and excited states are determined by

(17) $$n_{g}\left(r\right)=\frac{N}{1+e^{-2\delta (r)/kT}},$$

(18) $$n_{u}\left(r\right)=\frac{N}{1+e^{2\delta (r)/kT}}.$$

This becomes the initial state for interaction between both protons.

In the present system, tensile extension leads to extending the two coiled chains in the stretching direction at t<0, the two extended chains perfectly align, and *N* proton pairs with a single electron are formed at t=0. Saint–Venant’s principle (Axiom II) is always applicable to our system under tension, so that the external stress of this system is homogeneously dispersed into each pair of L- and R-protons at t≥0. Because the internal energy change due to the chain stretching process can be neglected, the macroscopic internal energy *U* is considered to be dominated by the interaction between protons at t≥0:

(19) $$U=N\langle \varepsilon \rangle +U_{0},$$

where $\langle \varepsilon \rangle$ is the averaged quantum interaction energy of each pair, and $U_{0}$ is the vibration and motion energy of the two chains. According to the “force theorem” [55,56], in addition to *Proposition 3*, the average energy $\langle \varepsilon \rangle$ is given by the mean value of the quantum interaction energy for each pair using the number fraction of each energy state as follows:

(20) $$\langle \varepsilon \rangle =\nu_{g}\left(r\right)\varepsilon_{g}+\nu_{u}\left(r\right)\varepsilon_{u},$$

where $\nu_{g}\left(r\right)=n_{g}\left(r\right)/N$ and $\nu_{u}\left(r\right)=n_{u}\left(r\right)/N$.

Since the total entropy change during deformation is given by −2Nklnz as described before, and $U_{0}$ is almost constant under isothermal conditions, the change of free energy F(r) of the present system has the form:

(21) $$\Delta F\left(r\right)=N(\nu_{g}\left(r\right)\varepsilon_{g}\left(r\right)+\nu_{u}\left(r\right)\varepsilon_{u}\left(r\right)-\varepsilon_{H})+2NkT\ln z.$$

As the extension proceeds, the fraction $\nu_{g}\left(r\right)$ rapidly approaches unity, and the free energy change can be simplified as

(22) $$\Delta F\left(r\right)/N≅\varepsilon_{g}\left(r\right)-\varepsilon_{H}+2kT\ln z≅\frac{1}{r}+\frac{J-K}{1+S}.$$

**Figure 5:**
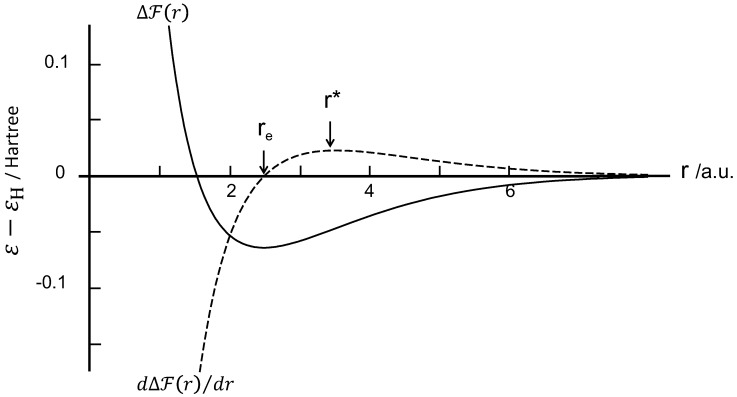
Free energy and attractive force in Hartree unit plotted against the interproton distance in atomic unit (a.u.).

The free energy of the present system is minimized at the minimum of $\varepsilon_{g}\left(r\right)$, which corresponds to an interproton distance $r_{e}$ of 0.132 nm (2.5 a.u.) and an energy depth $D\left(r_{e}\right)=\varepsilon_{g}\left(r_{e}\right)-\varepsilon_{H}$=−1.76 eV (–0.0648 Hartree). Figure 5 shows the free energy profile per proton pair at 300 K. It was found that the ΔF(r) is nearly insensitive to temperature and is almost identical to the $\varepsilon_{g}\left(r\right)$ profile. This is because the contribution of entropy is negligible at ambient temperatures in the present system. Furthermore, the orientation-induced crystallization leads to the energy loss being proportional to the length of the crystalline sequence *N*, given by $D(r_{e})N$. In other words, increasing of *N* leads to an additive increase of the cohesive force, suggesting a positive dependence of the stress with strain in the strain-hardening stage.

Orientation-induced crystallization of extended polymers can be interpreted as a spontaneous process, leading the system comprised of a pair of chains to achieve a free energy minimum under tensile elongation. This is the reason that orientation-induced crystallization proceeds without an activation barrier. Equation (21) illustrates that the free energy increases with increasing temperature, but the temperature sensitivity is negligibly small in this model. However, a rise in temperature does impede the polymer chains from attaining the fully stretched state under tension. Thus, it follows that the orientation-induced crystallization process is disturbed at higher temperatures.

On the basis of the force theorem, the attractive force function between two protons can be determined from dΔF(r)/dr, showing a maximum at $r^{*}$(=0.187 nm) at which the flexural point locates on the ΔF(r) curve. The attractive force profile is included in Figure 5. The $r_{e}$ is the equilibrium separation where the force is zero and the two chains separate spontaneously after reaching $r^{*}$. Then, the depth is $D(r^{*})=-1.29$ eV. In addition, the modulus $E_{Y}$ can be calculated from the second derivative of ΔF(r) with *r* as

(23) $$E_{Y}=\frac{1}{r^{2}}\frac{d^{2}}{dr^{2}}\Delta F\left(r\right){|}_{r\to r_{e}},$$

and yields $E_{Y}=293$ GPa, which is mostly in accordance with the experimental modulus (288±10 GPa) of polyethylene fibers [54]. Note that the $E_{Y}$ values are nearly independent of temperature in the present theoretical framework.

## 6. Conclusions

The significance of this work is to provide a novel physical concept for orientation-induced crystallization in order to explain a fundamental question of why the flexible polymers are crystallized under tension. In order to essentially understand the orientation-induced crystallization, we set up a simple coupled chains model as a small collection of a two-level quantum system and employed two axioms that (1) a crystallizable polymer molecule is expressed by a random-flight chain and (2) Poisson contraction is preserved through tension according to Saint–Venant’s principle. Accordingly, intermolecular interaction between two aligned chains can be postulated to be based on the hydrogen molecule ion H${}_{2}^{+}$, resulting in the origin for orientation-induced crystallization being the association of the interaction between extended chains due to a bonding (or ground state) orbital caused by a proton pair sharing one electron. When the tensile deformation proceeds, the resulting extended chains align along the elongation direction and adjacent extended chains become closer together. Then, the electron density around a proton bonded to a main chain carbon is spread out, resulting in minimization of a bonding orbital produced by the single electron system appearing between a pair of protons.

The present theory requires that polymer chains are surrounded by hydrogen atoms that are connected to the carbon atoms in the main chain. In order to widely characterize the orientation-induce crystallization for other flexible polymers, we need adjust the value of electron charges such that the ground-state potential curve is in accordance with the van der Waals potential function. For the purpose, we can propose a reduced electron charge *ξe*, where *ξ* is a factor of electron charge resulting from non-integral number of electron delivered from each proton [47]. In this work, *ξ* can be considered to be 1/2.

The spontaneous process leading to the minimum energy state is the origin for orientation-induced crystallization, and this spontaneous ordering process reflects that the orientation-induced crystallization has no activation barrier and does not need precursors. Therefore, this type of structural ordering process may be referred to as “solidification” rather than “crystallization”. The concept of solidification was first proposed by Fischer [57] to explain the crystallization process under large supercoolings for typical solid polymers in which their crystalline structure is formed by cooperative ordering of the stiffened segments that occurs by partial straightening of the coil sequences without a long-range diffusion process. The molecular relaxation due to viscosity may be introduced by replacing the Gaussian chain model with a Rouse–Bueche chain model [58,59] in which the bead friction is taken into account. This will make it possible to treat the crystallization and/or solidification under a non-perfect extension.
